# Connecting the Retina to the Brain

**DOI:** 10.1177/1759091414562107

**Published:** 2014-12-03

**Authors:** Lynda Erskine, Eloisa Herrera

**Affiliations:** 1 School of Medical Sciences, Institute of Medical Sciences, University of Aberdeen, Scotland, UK; 2 Instituto de Neurosciencias de Alicante, CSIC-UMH, San Juan de Alicante, Spain

**Keywords:** axon guidance, growth cone, optic chiasm, retinal ganglion cell, topographic mapping, visual system

## Abstract

The visual system is beautifully crafted to transmit information of the external world to visual processing and cognitive centers in the brain. For visual information to be relayed to the brain, a series of axon pathfinding events must take place to ensure that the axons of retinal ganglion cells, the only neuronal cell type in the retina that sends axons out of the retina, find their way out of the eye to connect with targets in the brain. In the past few decades, the power of molecular and genetic tools, including the generation of genetically manipulated mouse lines, have multiplied our knowledge about the molecular mechanisms involved in the sculpting of the visual system. Here, we review major advances in our understanding of the mechanisms controlling the differentiation of RGCs, guidance of their axons from the retina to the primary visual centers, and the refinement processes essential for the establishment of topographic maps and eye-specific axon segregation. Human disorders, such as albinism and achiasmia, that impair RGC axon growth and guidance and, thus, the establishment of a fully functioning visual system will also be discussed.

## Basic Anatomy of the Mammalian Visual System: From the Eye to the Cortex

**Figure 1.**
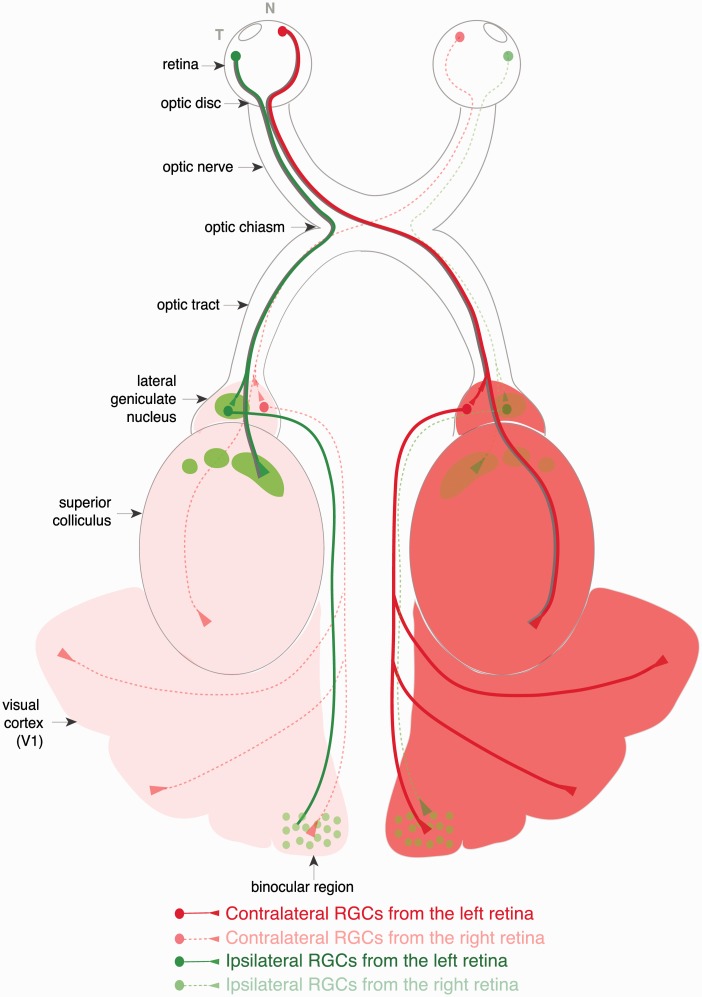
Schematic representation of the visual pathway in the mouse. RGC axons transverse the eye to exit at the optic disk. The axons then travel via the optic nerves to the optic chiasm where they cross or avoid the midline to project ipsilaterally or contralaterally in the optic tracts to the main visual targets: the LGN in the thalamus or the superior colliculus. Ipsilateral and contralateral projections form distinct patterns at the visual nuclei. While ipsilateral axons form a few confined patches at the rostral LGN and SC (green spots), the contralateral terminals fill the rest of the tissue (red/pink). Second-order relay neurons transmit visual information from the thalamus to the visual cortex. The visual cortex of the mouse is mostly monocular, but there is a small binocular region that receives thalamocortical input from both hemispheres.

The eyes together with their connecting pathways to the brain form the visual system. In the eye, the cornea bends light rays and is primarily responsible for focusing the image on the retina. The lens behind the cornea inverts the image top to bottom and right to left. The retina, the receptive surface inside the back of the eye, is the structure that translates light into nerve signals, and enables us to see under conditions that range from dark to sunlight, discriminate colors, and provide a high degree of visual precision. The retina consists of three layers of nerve cell bodies separated by two layers containing synapses made by the axons and dendrites of these cells. The back of the retina comprises the photoreceptors, the rods, and cones. The medial retinal layer contains three types of nerve cells, bipolar, horizontal, and amacrine cells. Bipolar cells receive input from the photoreceptors, and many of them feed directly into the retinal ganglion cells (RGCs). Horizontal cells connect receptors and bipolar cells by relatively long connections that run parallel to the retinal layers. Amacrine cells link bipolar cells and RGCs, the cells located in the inner retina. RGC axons pass across the surface of the retina and are collected in a bundle at the optic disk to leave the eye and form the optic nerve. There are approximately 20 RGC types that can be classified by morphological, molecular, and functional criteria. Each RGC type participates in distinct retinal circuits and projects to a specific set of targets in the brain (Coombs et al., 2007; Schmidt et al., 2011), including the main image-forming nuclei such as the lateral geniculate nucleus (LGN), the visual part of the thalamus, and the superior colliculus (SC), located in the roof of the midbrain, that coordinates rapid movement of the eye (Figure 1).

The optic axons from both eyes meet at the optic chiasm, which is located at the base of the hypothalamus. There, RGC axons from the nasal retina cross over to the opposite side of the brain (contralateral or commissural axons) while axons from the temporal retina turn to project to the same hemisphere as their side of origin (ipsilateral axons). Through evolution, the extent of binocular vision correlates with the frontalization of the eyes and, consequently, the proportion of ipsilateral axons (Jeffery and Erskine, 2005). In primates, the number of ipsilateral and contralateral RGC axons is approximately equal while in mouse, with more laterally positioned eyes, and hence a smaller degree of binocular overlap in the visual field, only around 3% to 5% of all RGCs are ipsilateral. In species without any binocular overlap in the visual field, such as chick, fish, and prematamorphic Xenopus tadpoles, ipsilateral projections are absent. After they pass the optic chiasm, ipsilateral axons, if present, combine with contralateral axons from the other eye to form the optic tracts (Figure 1).

A vast number of axons of the optic tract terminate in the LGN. The right LGN receives information from the left visual field (nasal left retina and temporal right retina), while the left LGN receives information from the right visual field (nasal right retina and temporal left retina; Figure 1). In carnivores and primates, the LGN consists of six layers. Layers 1, 4, and 6 correspond to information from the contralateral fibers of the nasal retina (temporal visual field); Layers 2, 3, and 5 correspond to information from the ipsilateral fibers of the temporal retina (nasal visual field). In rodents, the LGN lacks an obvious lamination pattern (Van Hooser and Nelson, 2006). Instead, retinal projections are organized into complementary nonoverlapping territories called eye-specific domains (Godement et al., 1984). The spatial relationships among the RGCs in the retina are maintained in their image-forming targets as an orderly representation (map) of visual space. Neighboring ganglion cells in the retina project to neighboring cells in the LGN and the SC, to form a continuous topographic map that reflects the visual image perceived by the retina (Rakic, 1976; Godement et al., 1984; Chalupa and Snider, 1998). From the dorsal LGN, second-order relay neurons, known as thalamocortical neurons, transmit visual information to the cortex where it is integrated with other sensory modalities to allow the brain to elaborate appropriated motor responses.

## RGCs Differentiation and Axonogenesis

### RGC Specification

Multipotent retinal progenitor cells give rise to all major cell types in the retina. Each cell type is born in a stereotypical and overlapping sequence, with RGCs the first to be generated in all vertebrates (Young, 1985; Holt et al., 1988; Wetts and Fraser, 1988; Turner et al., 1990). In mice, RGCs are born from embryonic day (E)11—postnatal day (P)0, with the peak at E14.5 (Drager, 1985; Hufnagel et al., 2010). In the human retina, RGC genesis begins around fetal week (Fwk) 5 and ends around Fwk 18 (Pacal and Bremner, 2014), whereas in fish and amphibians, new RGCs can be added throughout life.

Commitment of progenitors to a RGC fate occurs during, or just shortly after, the terminal cell division (McLoon and Barnes, 1989; Waid and McLoon, 1995; Prasov and Glaser, 2012; Pacal and Bremner, 2014) and is controlled by a combination of intrinsic factors and cell–cell signals. Differentiation of RGCs begins in the central retina and spreads peripherally in a roughly concentric fashion (Drager, 1985; Hu and Easter, 1999; McCabe et al., 1999; Neumann and Nuesslein-Volhard, 2000; Martinez-Morales et al., 2005; Hufnagel et al., 2010). The initial central patch of RGC differentiation is triggered by fibroblast growth factors FGF3 and FGF8 and released by species-specific organizing centers in the optic stalk and neural retina (Masai et al., 2000; Martinez-Morales et al., 2005). Delta-Notch signaling plays an important role in determining whether retinal progenitors will differentiate or not. Blocking Notch or Delta signaling in retinal progenitors results in premature differentiation and a subsequent increase in the number of RGCs due to the bias toward RGC production during early retinogenesis (Austin et al., 1995; Henrique et al., 1997). Sfrps (secreted frizzled-related proteins) negatively regulate Notch-signaling in the retina, through inhibition of the metalloprotease Adam10 essential for Notch activity. Thus, in compound mouse mutants for *Sfrp1* and *Sfrp2*, retinal neurogenesis is disrupted (Esteve et al., 2011).

Notch signaling regulates retinal neurogenesis in part through controlling the expression of genes essential for cell fate specification, such as *Atoh7* (Maurer et al., 2014). The bHLH proneural gene *Atoh7* (previously known as *Math5*) is essential for commitment toward the RGC fate. Atoh7 is expressed in retinal progenitors coincident with the onset of RGC differentiation. In mice lacking *Atoh7*, few RGCs are generated, with progenitor cells instead adopting an amacrine or cone photoreceptor fate (Brown et al., 2001; Wang et al., 2001). Downstream targets of Atoh7 include the POU-domain transcription factor Brn3b (POU4F2) and the LIM-homeodomain transcription factor Islet-1 (Isl1; Wang et al., 2001; Yang et al., 2003). Brn3b and Isl1 are expressed by RGCs during or shortly after their terminal cell division (Prasov and Glaser, 2012; Pacal and Bremner, 2014), and act in parallel pathways, to activate genes essential for RGC morphological differentiation and survival (Erkman et al., 2000; Pan et al., 2008). Brn3b is expressed by about 80% of nascent RGCs and the related factors Brn3a (Pou4f1) and Brn3c (Pou4f3) in ∼80% and ∼20% of RGCs, respectively. In mouse, expression of Brn3a and Brn3c in RGCs begins approximately 1 day after Brn3b, with each factor expressed in overlapping subsets of cells (Xiang et al., 1995; Pan et al., 2005). Although initially thought to be functionally equivalent (Pan et al., 2005), elegant studies in mice using *Brn3a* and *Brn3b* conditional alleles have demonstrated distinct roles for Brn3a and Brn3b in control of RGC subtype identity (Badea et al., 2009; Shi et al., 2013).

A combination of intrinsic and extrinsic signals also plays an important role in driving the RGC differentiation wave across the retina. Neurog2, a bHLH transcription factor highly related to Atoh7, is a key factor regulating the spatial and temporal progression of retinal neurogenesis. In mice lacking *Neurog2*, the neurogenic wave front stalls temporally resulting in disruption of RGC genesis and smaller retinas. The reinstatement of neurogenesis in *Neurog2* mutants correlates with onset of expression of *Ascl1*, a later expressed bHLH transcription factor, suggesting functional redundancy between Neurog2 and Ascl1 (Hufnagel et al., 2010). FGF1 and the classical morphogen Sonic hedgehog (Shh) have also been implicated in driving the propagation of the RGC differentiation wave across the retina (McCabe et al., 1999; Neumann and Nuesslein-Volhard, 2000). However, the role of Shh remains controversial and may be species specific. In all species, Shh is released by newly generated RGCs and can act on undifferentiated neural progenitor cells to regulate differentiation. Genetic or pharmacological disruption of hedgehog signaling in zebrafish retinas has no impact on differentiation of the first central patch of RGCs but neurogenesis fails to spread further, demonstrating a positive role for hedgehog-signaling in propagating RGC differentiation in fish (Neumann and Nuesslein-Volhard, 2000). However, studies in chick and mouse have failed to replicate this finding and instead indicate that Shh is a negative regulator of RGC genesis in these species (Zhang and Yang, 2001; Wang et al., 2005). The rational for this species-specific difference in Shh function in RGC differentiation is not clear currently but may reflect differences in the level of Shh signaling or the length of the neurogenic period.

Other factors released by RGC themselves modulate the production and survival of new RGCs, thereby acting in a feedback loop to control RGC number (Waid and McLoon, 1998; Gonzalez-Hoyuela et al., 2001). The list of these factors may be not complete but includes vascular endothelial growth factor-A (VEGF-A), neural epidermal growth factor-like (NEL), and growth differentiation factor 11 (Gdf11; Kim et al., 2005; Hashimoto et al., 2006; Nakamoto et al., 2014). Further studies will be required to identify the full repertoire of extrinsic signals essential for RGC genesis and their interplay with the cell-intrinsic factors important for the commitment to a RGC fate.

### Morphological Differentiation of RGCs

**Figure 2.**
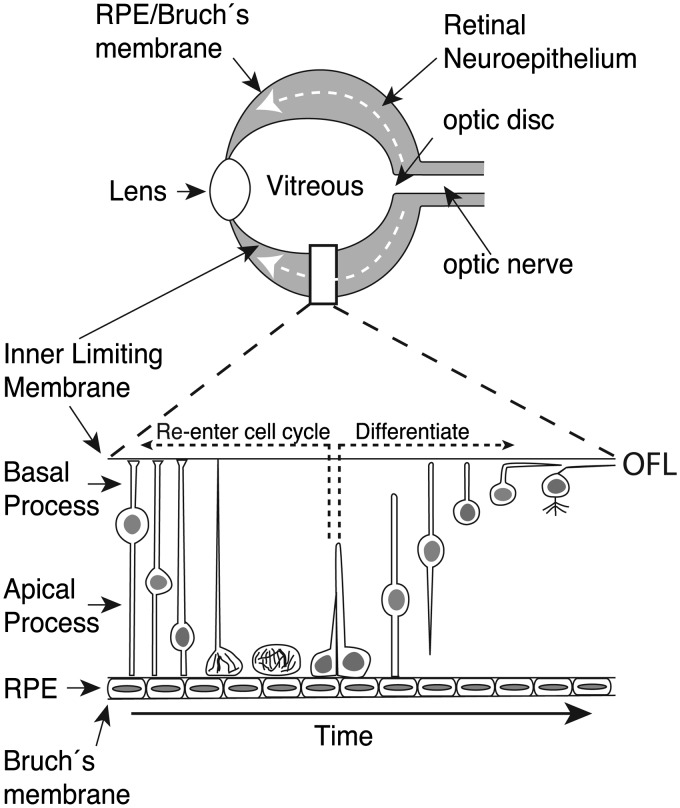
Schematic representation of RGC differentiation. Neuronal differentiation in the retina proceeds in central-to-peripheral waves (white-dotted arrows in retina schematic). The sequence of RGC differentiation is shown in the lower panel. Retinal progenitor cells undergo interkinetic nuclear migration during the cell cycle (left hand side of lower panel) and divide adjacent to the RPE. Following cell division, the daughter cells can either reenter the cell cycle (black-dotted arrow) or exit the cell cycle and form a differentiated neuron (right hand side of lower panel). Nascent RGCs retract their apical process and translocate their nucleus into the RGC layer at the inner (vitreal) surface of the retina. As the nucleus translocates, an axon emerges that extends into the optic fiber layer (OFL) where it grows toward the optic disk. Neuronal polarity is regulated by extracellular matrix molecules of the inner limiting membrane and Bruch’s membrane.

Nascent RGCs undergo their terminal division adjacent to the retinal pigmented epithelium (RPE; ventricular) surface, forming spherical cells with no obvious polarity. These apolar cells extend a basal process through the thickness of the retina toward the vitreal surface, generating cells with a bipolar morphology attached to the inner and outer limiting membranes of the retina. The RGC nucleus then migrates through the basal process toward the inner surface of the retina. Whilst this migration is occurring, an axon extends, usually from the basal process, into the optic fiber layer. Finally, the bipolarly shaped cell breaks the attachment of its apical process with the ventricular surface, resulting in a morphologically distinguishable RGC located within the RGC layer at the inner surface of the retina. Formation of dendrites occurs after axon initiation but before target innervation (Hinds and Hinds, 1974; Holt, 1989; McLoon and Barnes, 1989; Snow and Robson, 1994; Zolessi et al., 2006; Figure 2).

Slit-Robo signaling plays an essential role in regulating apical process retraction in differentiating RGCs. In zebrafish with morpholino-induced knockdown of *slit1b* or *robo3*, retraction of the apical process and nuclear migration are delayed (Zolessi et al., 2006; Wong et al., 2012). On the other hand, dominant-negative N-cadherin constructs induce premature apical process detachment (Wong et al., 2012). Because Slit-signaling is a known negative regulator of N-cadherin-mediated adhesion (Rhee et al., 2002), it is likely that Slit-signaling induces apical process retraction through inhibiting N-cadherin function.

Time-lapse confocal imaging of RGC differentiation in zebrafish embryos has demonstrated that axon emergence occurs rapidly and in a highly directed fashion (Zolessi et al., 2006). Unlike in culture, where multiple transient processes are extended prior to development of the axon, *in vivo*, axons emerge in the absence of other neurites. Axon initiation occurs independently of apical process retraction (Zolessi et al., 2006) and is regulated by a range of extrinsic signals. Blocking cadherin, NF-protocadherin, or β-integrin signaling in *Xenopus* RGCs impairs axon initiation and outgrowth, demonstrating an important role in initial axon extension for cell adhesion and integrin signaling (Lilienbaum et al., 1995; Riehl et al., 1996; Piper et al., 2008). Cytoskeletal rearrangement is also important as evidenced by blocking of the Rho GTPase Rac1 with a dominant-negative construct that inhibits axonogenesis (Ruchhoeft et al., 1999). The initial polarity of axon outgrowth is controlled, at least in part, by signals from the retinal basal lamina. In zebrafish *nok* and *has* mutants with disrupted neuroepithelial polarity and basal lamina development, many RGCs fail to polarize normally, extending axons aberrantly along the outer (RPE) surface of the retina (Zolessi et al., 2006). Similarly, digesting chondroitin sulfate proteoglycans (CSPG), components of the retinal basal lamina, or adding exogenous free CS chains to cultured rat retinas can induce a complete reversal in RGC polarity, with cell bodies and axons located adjacent to the RPE (Brittis et al., 1992; Brittis and Silver, 1994).

## Axon Growth and Orientation Within the Retina and Exit From the Eye

### Growth Into the Optic Fiber Layer

**Figure 3.**
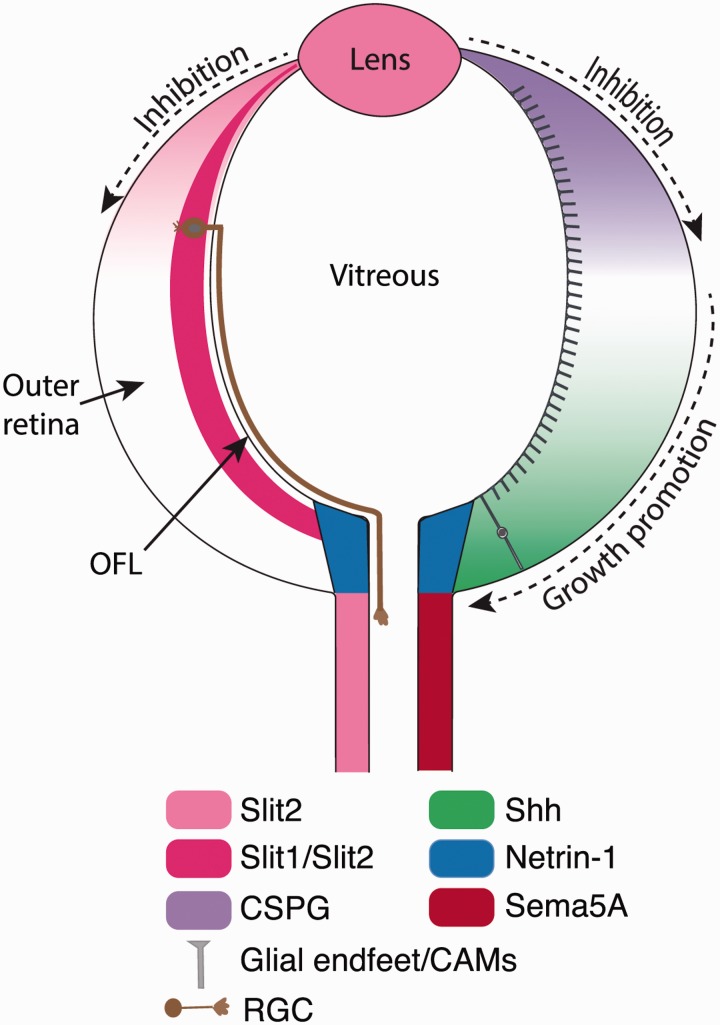
Expression pattern of key molecules that direct intraretinal RGC axon guidance. RGC axons express Robo receptors and are responsive to Slit-signaling from shortly after axonogenesis. Slit1 and Slit2 present in the inner retina prevent RGC axon growth into the outer retina, helping ensure that axons extend directly into the optic fiber layer (OFL) at the inner surface of the retina. Within the optic fiber layer, the endfeet of neuroepithelial cells (glial endfeet) and cell adhesion molecules (CAMs) provide a permissive substrate. Growth of RGC axons toward the optic disk is highly directed from the outset and controlled by inhibitory gradients of Slit2 and CSPGs, highest in the retina periphery, and a centrally located attractive gradient of Shh that helps drive growth centrally (black-dashed arrows). Whether these gradients of growth-promoting and inhibitory molecules overlap within the retina or are confined to distinct domains is not known currently. Netrin-1, expressed by the optic disk glia, is essential for growth out of the eye. Inhibitory molecules, such as Slit2 and Sema5A, help restrict the RGC axons to the optic nerve once they exit the eye. In the interest of clarity, most molecules are shown only on one side of the retina but will be symmetrically distributed *in vivo*.

Following their initiation, RGC axons extend toward the optic disk, their exit point from the eye, within a narrow zone at the inner (vitreal) surface of the retina—the optic fiber layer. Growth of retinal axons is restricted to the optic fiber layer through a combination of the growth-promoting properties of the glial endfeet present in this region, and the inhibitory properties of the outer retinal layers (Stier and Schlosshauer, 1995; Figure 3). Purified glial endfeet provide an excellent substrate for RGC axon outgrowth and are required for growth of retinal axons within the optic fiber layer (Stier and Schlosshauer, 1995). The cell adhesion molecule, NCAM (neural cell adhesion molecule), localizes to the glial endfeet and contributes to their growth-promoting activity (Halfter et al., 1987; Brittis et al., 1995). Conversely, Slit proteins are a component of the inhibitory activity of the outer retina (Thompson et al., 2006b). RGC axons express the Slit-receptor Robo2 (Plachez et al., 2008; Thompson et al., 2009) and are responsive to inhibitory Slit signaling shortly after axonogenesis (Thompson et al., 2006b). In mice lacking *slit1* and *slit2*, the only murine Slit family members expressed within the developing retina (Erskine et al., 2000; Niclou et al., 2000), or their receptor *robo2*, fascicles of RGC axons extend aberrantly through the outer layers of the retina (Thompson et al., 2006b, 2009). Because only a small subset of axons project into the outer retina in the absence of Slit-Robo signaling, additional inhibitory factors are likely involved in preventing growth away from the optic fiber layer. Currently, the identity of these additional signals is not known.

### Growth Toward the Optic Disk

From the outset, growth within the optic fiber layer is highly organized and directed straight toward the optic disk. Surprisingly, RGC axons located aberrantly in the outer retina in compound *Slit1/2* or *Robo2* mouse mutants (Thompson et al., 2006b) or zebrafish *nok* mutants with defective retinal polarity (Zolessi et al., 2006) still grow toward the optic disk and exit the eye. This suggests that the cues for disk-directed growth are not restricted exclusively to the optic fiber layer. However, no evidence has been found for a long-range attractant controlling disk-directed growth. If small regions of the retinal neuroepithelium are rotated 180°, RGC axons grow away from the optic disk at a rate equal to axons growing in the correct direction within the nonrotated epithelium (Brittis and Silver, 1995; Halfter, 1996). Moreover, transplanting the optic disk to ectopic locations does not reorient nerve growth (Halfter, 1996). Instead, the highly organized, radial growth of RGC axons appears to depend on the combined action of local permissive cues within the neuroepithelium and inhibitory signals that prevent growth peripherally (Figure 3).

Prior to axonogenesis, the retinal neuroepithelium is inhibitory to axonal growth (Halfter, 1996). Because retinal neurogenesis is initiated in the central retina and spreads peripherally, newly differentiated RGCs are bordered by a peripheral inhibitory zone and permissive central region, helping ensure that axon growth is directed centrally from the outset. In rodents, the inhibitory properties of the retinal neuroepithelium correlate with a centrally–peripherally receding wave of CSPG. As the CSPG wave passes over the undifferentiated neuroepithelial cells, this helps control the timing of RGC genesis and, through inhibitory signaling, directs axon growth away from the retinal periphery, straight toward the optic disk (Brittis et al., 1992). Inhibitory factors, such as Slit2 secreted by the lens, also help ensure that RGC axon growth is directed centrally from the outset (Ohta et al., 1999; Thompson et al., 2006b). In chick retinas, inhibitory factors under the control of the transcription factor Zic3 have also been demonstrated to play a role in ensuring axon growth is directed centrally (Zhang et al., 2004). However, the identity of these Zic3-regulated inhibitory factors has not been determined.

Acting in concert with the inhibitory forces *pushing* the RGC axons away from the retinal periphery are a range of cell adhesion molecules such as L1, NrCAM, and BEN (also called DM-GRASP/SCI/Neurolin) that help drive growth centrally. Blocking the function of these molecules in chick, fish, or rodent retinas impairs the tight bundling together of RGC axons and the disk-directed growth of some axons (Brittis et al., 1995; Ott et al., 1998; Weiner et al., 2004). In zebrafish, the chemokine SDF-1, acting through its receptor CXCR4 on the RGC axons, is required for normal disk-directed growth. Morpholino knockdown of *sdf-1a* or *cxcr4b* results in aberrant axon growth within the retina, with axons often extending away from the optic disk (Li et al., 2005). Another factor implicated in promoting disk-directed growth is Shh. As mentioned above, Shh is expressed in a dynamic high central-low peripheral retinal gradient that corresponds to the pattern of RGC differentiation. *In vitro*, Shh can act directly on RGC axons to either promote or inhibit RGC axon outgrowth depending on its concentration (Kolpak et al., 2005). Both overexpression and loss of Shh function in chick retinas results in a complete loss of disk-directed growth (Kolpak et al., 2005). Similarly, in mice, blocking Shh signaling in RGCs by electroporation of a Shh-insensitive Ptc1 (Patched-1) receptor impairs RGC axon growth in the region of the optic disk, with many axons growing parallel to the disk or into the peripheral retina (Sanchez-Camacho and Bovolenta, 2008). However, the signaling mechanisms underlying the response of RGCs to Shh are unclear. Ptc1 localizes to RGC axons and has been implicated in mediating the inhibitory response of RGCs to high concentrations of Shh (Trousse et al., 2001; Sanchez-Camacho and Bovolenta, 2008). However, the *in vivo* role for Ptc1 in RGC axons has not been established. Protein kinase Cα (PKCα) and integrin-linked kinase (ILK) are required for the inhibitory signaling of high concentrations of Shh in RGC axons (Guo et al., 2012), but this signaling pathway is not required for disk-directed growth (Guo et al., 2012). *Hip1* (hedgehog-interacting protein) is also expressed by all RGCs, but it is unclear whether it plays a role in retinal axon directionality.

Transcription factors such as Brn3b and Pax6, in addition to playing a role in RGC differentiation, are also involved in controlling the growth of RGC axons toward the optic disk (Erkman et al., 2000; Manuel et al., 2008). In mice lacking *Brn3b* or expressing multiple copies of human PAX6 (*Pax77^+/+^* mice), RGC axon fasciculation is impaired, with many axons following abnormal trajectories within the retina, and, in the case of *Brn3b* mutants, failing to reach the optic disk (Erkman et al., 2000; Manuel et al., 2008). Several target genes regulated by Brn3b have been identified including the actin-binding protein abLIM, which plays an essential role in controlling RGC axon fasciculation and disk-directed growth (Erkman et al., 2000).

### Growth Out of the Eye

Once axons reach the optic disk, they make an approximately 45° change in their direction of growth to exit the eye (Figure 3). Normal development and patterning of the optic disk is essential for growth out of the eye. In mice mutants with disrupted development of optic disk glial cells or optic fissure closure, for example, heterozygous *krd* (kidney and retinal defects; Otteson et al., 1998) or *bst* (belly spot and tail; Rice et al., 1997) mutants, conditional mutants lacking *shh* in RGCs (Dakubo et al., 2003) or *tbx2* mutants (Behesti et al., 2009), many RGC axons fail to exit the eye. Similarly, in BMP receptor 1B mouse mutants, with disrupted guidance cue expression at the optic disk, exit of RGC axons from the eye is impaired (Liu et al., 2003).

A key signaling molecule essential for RGC axon growth out of the eye is Netrin-1, acting through its receptor DCC (deleted in colorectal carcinoma) on the RGC axons (Deiner et al., 1997). Netrin-1 localizes to the optic disk glia (Figure 3) and is normally growth promoting and attractive for RGC axons (Deiner et al., 1997; de la Torre et al., 1997). In mice lacking Netrin-1 or DCC, retinal axons grow normally toward the optic disk but fail to exit the eye resulting in optic nerve hypoplasia (Deiner et al., 1997). This suggests that, in contrast to the spinal cord where Netrin-1 acts as a long-range attractant for commissural axons (Kennedy et al., 1994), Netrin-1 acts locally within the retina to direct guidance at the optic disk. If Netrin-1 is attractive for RGC axons, why do they turn away from the high Netrin-1 levels at the surface of the optic disk into the optic nerve? An elegant series of experiments in *Xenopus* demonstrated that changes in growth cone cAMP levels, triggered by contact with the extracellular matrix protein laminin-1, convert Netrin-mediated attraction into repulsion, driving growth out of the eye (Hopker et al., 1999). At the entry into the optic disk, laminin-1 localizes to the retinal surface. By reducing growth cone cAMP levels, this induces inhibitory signaling from the Netrin-1 colocalized in this region, helping drive the axons into deeper regions of the optic disk. Netrin-1 function and localization within the eye may also be modulated by Heparan sulfate proteoglycans (HSPGs). *In vitro*, the growth-promoting activity of Netrin-1 is reduced in HS-deficient RGC axons, and mice with defective HS synthesis in the retina display a range of RGC axon guidance errors, including impaired growth out of the eye (Ogata-Iwao et al., 2011). Reverse signaling by EphBs acting as inhibitory guidance cues also helps target a subset of dorsal but not ventral RGC axons to the optic disk and out of the eye (Birgbauer et al., 2000).

## Guidance at the Optic Chiasm

### Cellular and Molecular Determinants of the General Path Toward and Through the Optic Chiasm

Once RGC axons exit the eye, they grow amongst the intrafascicular glial cells of the optic nerve (Silver, 1984). Sema5A and Slit2 are expressed by the optic nerve glial cells and, through inhibitory signaling, help restrict the RGC axons to the optic nerve and ensure they remain tightly bundled together (Plump et al., 2002; Oster et al., 2003; Figure 3). The transcription factor Vax1 is also expressed by the optic nerve glia as well as in the ventral diencephalon and is required for growth of retinal axons from the optic nerve to form the chiasm. In mice lacking *Vax1*, RGC axons stall as they enter the brain and fail to form the chiasm (Bertuzzi et al., 1999). At the site where the axons stall in *Vax1* mutants, expression of the growth-promoting molecule Netrin-1 is lost whereas Slit1 expression is maintained. Initially, it was thought that these alterations in guidance cue expression may create an environment that is inhibitory to axon extension and underlie the failure of RGC axons to grow into the brain in *Vax1* mutant mice (Bertuzzi et al., 1999). Recently, however, it has been shown that Vax1 protein is secreted by ventral diencephalic cells and, independent of its transcription factor activity, can promote RGC axon outgrowth. This raises the intriguing possibility that Vax1 may act directly on the RGC axons growth cones as a guidance cue essential for optic chiasm formation (Kim et al., 2014).

**Figure 4.**
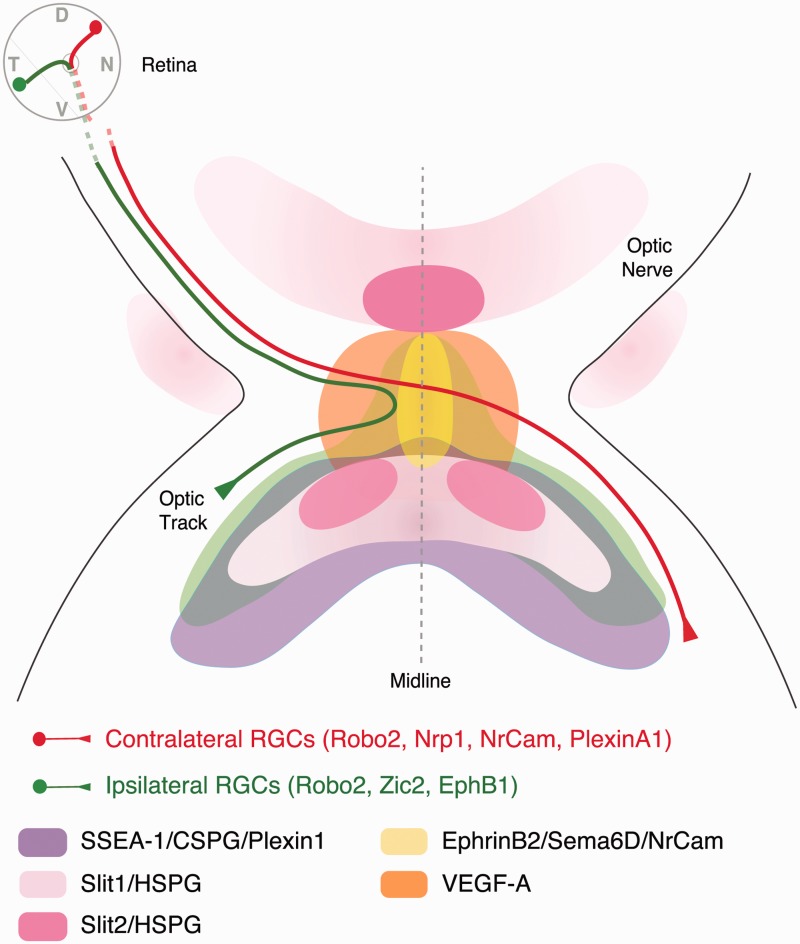
Expression pattern of key molecules that direct RGC axon navigation at the optic chiasm of mice. RGC axons expressing Robo receptors reach the optic chiasm region starting at E10-E11. There diffusible Slit molecules shape a repulsion-free corridor that delineates the path the RGC axons grow through. SSEA-1-positive neurons expressing CSPG are also located at the ventral diencephalon helping to establish the general optic chiasm path. Axonal divergence between ipsilateral axons (green), arising at the ventrotemporal retina, and contralateral axons (red), coming from the rest of the retina, occurs at the midline. Ipsilateral axons express EphB1, which is induced by the transcription factor Zic2. At the midline, ipsilateral axons are repelled by ephrin-B2 that is expressed by glial cells. As a consequence of the EphB1/ephrin-B2 interaction, ipsilateral axons turn about 90° to project in the same hemisphere. Contralateral axons do not express EphB1 and ignore Ephrin-B2. In contrast, because they express Neuropilin1, contralateral axons are attracted by VEGF-A also expressed at the midline. Positive interactions between NrCAM and PlexinA1 on contralateral axons and PlexinA1 and NrCAM together with Sema6D at the midline also help promote midline crossing. D = dorsal; N = nasal; V = ventral; T = temporal.

Once the axons reach the chiasm, the organization of the glial cells changes from intrafascicular to radial (Colello and Guillery, 1992; Marcus et al., 1995). A population of diencephalic neurons expressing the cell surface antigens SSEA-1 (Stage-specific embryonic antigen-1) and CD44 are also present at the murine chiasm. These neurons form an inverted V-shaped array that defines the posterior border of the optic chiasm and extend as a narrow raphe along the midline (Sretavan et al., 1994; Marcus et al., 1995; Figure 4). Ablation of these neurons results in RGC axon stalling as they enter the brain and failure of chiasm formation (Sretavan et al., 1995).

As they extend toward and through the optic chiasm, the RGC axons are bounded by inhibitory Slit signals that play an essential role in defining the precise path followed by the axons (Figure 4). In mice or zebrafish lacking Slit-Robo signaling, many RGC axons stray away from their normal pathway, including crossing the midline in ectopic anterior locations, extending in increased numbers into the contralateral optic nerve, and wandering posteriorly along the midline (Fricke et al., 2001; Plump et al., 2002; Plachez et al., 2008). Slit signaling at the chiasm is modulated by HSPGs and intracellular cAMP levels. HSPGs both help localize Slits to the extracellular matrix (Wright et al., 2012) and are required in RGC axons for growth cone responses to Slits (Piper et al., 2006; Pratt et al., 2006). In mouse or zebrafish mutants with disrupted HS biosynthesis (*Ext1*: Inatani et al., 2003; *Hs6st1, Hs2st*: Pratt et al., 2006; Conway et al., 2011; *dak/ext2;box/extl3*: compound mutants Lee et al., 2004), RGC axons make pathfinding errors at the optic chiasm that, in part, phenocopy *slit* and *robo2* mutants. The chemokine SDF-1 has no effect on axon outgrowth by itself. However, SDF-1 reduces the inhibitory activity of multiple guidance cues, including Slits, by acting through its receptor CXCR4 to elevate growth cone cAMP levels (Chalasani et al., 2003). SDF-1 can modulate Slit-Robo signaling *in vivo*, and blocking either SDF-1 signaling or its downstream target ADCY8 (adenylate cyclase 8) rescues RGC axon pathfinding errors in zebrafish mutants with partial loss of Robo2 function (Chalasani et al., 2007; Xu et al., 2010). Metalloprotease-induced modulation of guidance responses, including Slit-signaling, has also been implicated in regulating RGC axon pathfinding at the optic chiasm and tract (Webber et al., 2002; Hehr et al., 2005).

In addition to Slits and their modulators, other factors including Shh and CSPGs have been implicated in restraining the path followed by RGC axons as they grow through the optic chiasm. Shh is expressed along the entire central nervous system (CNS) midline, except for a small gap where the optic chiasm develops. Thus, RGC axons normally extend across the midline through a Shh-free region, with high levels of Shh bordering the chiasm both anteriorly and posteriorly (Marcus et al., 1999; Trousse et al., 2001). This gap in Shh expression is essential for RGC axon to progress normally across the midline. In Pax2 mutants, or zebrafish *fgf8/ace* mutants with disrupted *Pax2* expression, Shh is expressed along the entire CNS midline with no gap at the chiasm region. In these mutants, RGC axons can no longer grow across the midline but instead project ipsilaterally (Torres et al., 1996; Macdonald et al., 1997; Shanmugalingam et al., 2000). Consistent with this, both *in vitro* assays and gain-of-function experiments in chick embryos have demonstrated that Shh can act directly on contralaterally projecting RGC axons to provide inhibitory signals (Trousse et al., 2001; Sanchez-Camacho and Bovolenta, 2008). Blocking Shh signaling in mouse embryos *in vivo* during the period when the optic chiasm develops has no gross impact on retina or diencephalon patterning, but results in RGC axon pathfinding defects, including defasciculation, an increase in the area occupied by the contralateral axons as they cross the midline, and increased numbers of axons extending ipsilaterally and into the contralateral optic nerve (Sanchez-Camacho and Bovolenta, 2008). Thus, similar to Slits, Shh bordering the optic chiasm acts normally to help constrain the RGC axons to their correct pathway as they extend through the optic chiasm. CSPGs are expressed by the SSEA-1-positive neurons that define the posterior border of the chiasm. Treatment of cultured retina–ventral diencephalon slices with chondroitinase ABC results in disorganized growth at the optic chiasm, including axons crossing in aberrant locations, and wandering away from their normal path (Chung et al., 2000). Whether CSPGs act directly on the RGC axons to direct growth at the chiasm, or through modulation of other guidance signals, for example, Slits or Shh, remains to be determined.

### Molecular Mechanisms of Midline Crossing at the Optic Chiasm

In all species, the majority of RGC axons project contralaterally at the optic chiasm. However, for many years, the mechanisms that control crossing at the chiasm remained elusive. Chemoattractants, such as netrin-1 (Kennedy et al., 1994) and Shh (Charron et al., 2003), essential for attracting commissural axons toward the spinal cord midline, are not expressed at the diencephalic midline (Deiner and Sretavan, 1999; Marcus et al., 1999; Trousse et al., 2001). Moreover, as outlined above, rather than being attractive for contralateral RGC axons, Shh actually inhibits their outgrowth (Trousse et al., 2001; Sanchez-Camacho and Bovolenta, 2008). This led to the idea that midline crossing at the chiasm may be a default process rather than requiring active signaling. We now know that this is not the case and the action of many molecules is essential to assure midline crossing (Figure 4).

One of the first proteins shown to be essential for midline crossing at the chiasm was the cell-adhesion molecule NrCAM. NrCAM is expressed both by contralaterally projecting RGCs and the ventral midline glial cells (Williams et al., 2006). In mice lacking NrCAM, a small subset of late-generated contralaterally projecting RGCs fails to project axons across the midline. Most contralateral axons, however, crossed normally. Moreover, *in vitro* experiments demonstrated that, when acting alone, NrCAM is required exclusively within the retina for promotion of RGC axon growth on chiasm cells (Williams et al., 2006). Subsequent experiments, however, demonstrated that NrCAM acts in a tripartite complex with Sema6D coexpressed with NrCAM on the midline radial glia and Plexin-A1 on the SSEA-1-positive neurons to provide growth-promoting signals for contralateral RGC axons. All three molecules are required in combination for efficient crossing at the optic chiasm (Kuwajima et al., 2012).

Surprisingly, the first midline factor shown to act as a chemoattractant and promote growth of contralateral axons at the chiasm was not a molecule with a known role in axon navigation, but rather a neuropilin-1 (NRP1) binding isoform of the classical vascular growth factor VEGF-A (Erskine et al., 2011). Strong expression of VEGF-A is present at the chiasm midline, whereas NRP1 is expressed by contralateral RGCs. *In vitro*, VEGF-A provides growth-promoting and chemoattractive signals for contralateral RGC axons and is required *in vivo* for contralateral growth. In mice lacking either *Nrp1* or expressing only the VEGF_120_ isoform that cannot signal through NRP1, the size of the ipsilateral projection is increased substantially. This increase in ipsilateral projections is due to a failure of presumptive contralateral axons to cross the midline.

NRP1 has also been shown to be important for contralateral RGC axon growth in zebrafish (Dell et al., 2013). However, in this species, the identified ligand is a classical NRP1-signaling partner in the nervous system, Semaphorin (Sema) 3D (Sakai and Halloran, 2006). Whether VEGF-A is also essential for chiasm development in zebrafish has not yet been investigated. In contrast to mice, where Semaphorin-signaling through Neuropilins is not essential for chiasm development (Erskine et al., 2011), knockdown of *sema3d* in zebrafish results in axons stalling in the proximal optic tract and aberrant ipsilateral projections (Sakai and Halloran, 2006). *Sema3d* is expressed anterior, posterior, and dorsal to the developing optic chiasm and is not required for midline crossing per se, but for growth from the chiasm into the proximal optic tract (Sakai and Halloran, 2006). Whether Sema3D provides growth-promoting or inhibitory signals to RGC axons remains unclear (Sakai and Halloran, 2006; Dell et al., 2013). However, in its absence, RGC axons stall in the proximal optic tract, or repeatedly advance and retract between the midline and the proximal optic tract, with some axons misrouting into the ipsilateral pathway (Sakai and Halloran, 2006). Mice lacking GAP-43 (growth-associated protein 43) display a similar defect in RGC axon growth from the chiasm into the proximal optic tract (Strittmatter et al., 1995). GAP-43 in an abundant intracellular growth cone protein that becomes phosphorylated by PKC in response to extracellular guidance cues, resulting in modulation of the growth cone actin cytoskeleton. Although expressed both by RGC growth cones and cells along the optic pathway, transplantation experiments have demonstrated that GAP-43 is required autonomously in RGC growth cones for normal interaction with the diencephalic cells lining the proximal optic tract. In the absence of GAP-43, RGC axons stall as they enter the optic tract resulting in axons backing up into the chiasm and randomized routing either ipsilaterally or contralaterally (Kruger et al., 1998; Sretavan and Kruger, 1998; Zhang et al., 2000). Thus, proper formation of the contralateral optic tract requires factors to both promote midline crossing, such as VEGF-A, as well as molecules that permit growth from the chiasm into the proximal tract.

NRP1 expression in different types of neurons, including zebrafish RGCs and mammalian olfactory sensory neurons, is regulated at the transcriptional level by intracellular cAMP levels (Imai et al., 2006; Dell et al., 2013). Decreasing RGC cAMP levels in zebrafish through expression of a dominant-negative (DN) G-protein subunit (DNGα_s/olf_) reduces levels of *Nrp1a* and *Nrp1b* mRNA in RGCs. This results in RGC axons guidance errors that phenocopy knockdown of *Nrp1a*, or its known chiasm ligands in zebrafish, *sema3d*, and *sema3e* (Dell et al., 2013). Thus, cAMP can act in two distinct ways to modulate RGC axon pathfinding. First, cAMP can act rapidly to alter the response of growth cones to guidance cues, changing attractive responses to repulsion and vice versa (Song et al., 1998; Hopker et al., 1999; Chalasani et al., 2003; Chalasani et al., 2007; Xu et al., 2010). Second, cAMP can act over a longer time period to regulate the transcription of genes encoding axon guidance receptors, such as NRP1, controlling the ability of the growth cone to respond to specific environmental signals (Dell et al., 2013).

### Molecular Mechanisms of Midline Avoidance at the Optic Chiasm

In mammals and other species with binocular vision, such as postmetamorphic *Xenopus*, a population of RGCs project to the ipsilateral hemisphere instead of crossing the midline at the optic chiasm. Ipsilateral RGC axons reach the midline but once there they are repelled by repulsive signals from glia and other cells in the ventral diencephalon. One of the families of guidance molecules implicated in midline avoidance are the membrane-anchored proteins, ephrins, and their tyrosine kinase receptors, the Ephs. Ipsilateral RGC axons express high levels of EphB1, while glial cells at the midline express its ligand ephrinB2 (Williams et al., 2003; Figure 4). When ipsilateral axons approach chiasm glial cells, the binding of EphB1 to ephrinB2 mediates a repulsive response that provokes the turning of ipsilateral axons away from the midline (Williams et al., 2003; Petros et al., 2009). Further analysis of EphB and ephrin-B mutant mice has demonstrated that, despite the coexpression of EphB2 in the ipsilaterally projecting retinal axons, EphB1 is the preferred receptor of ephrin-B2 (Chenaux and Henkemeyer, 2011). The expression of EphB1 in ipsilateral RGCs is controlled by the zinc finger transcription factor Zic2 (Garcia-Frigola et al., 2008), a main determinant of axon midline avoidance in the CNS (Herrera et al., 2003; Escalante et al., 2013). The transcription factor Islet-2 (Isl2) is expressed by a subset of contralateral RGCs throughout the retina but is required specifically within the region of binocular overlap in the visual field (ventrotemporal retina in mice) to repress the expression of Zic2/EphB1 (Pak et al., 2004). Its role in the contralateral RGCs outside the ventrotemporal retina is not known.

Shh-mediated signaling through one of its receptors Boc is also implicated in the generation of the ipsilateral projection. Boc is expressed in ipsilateral but not contralateral RGCs and, as stated above, Shh is secreted by contralateral RGCs and also by the ventral diencephalon midline, except where the optic chiasm develops (Trousse et al., 2001; Sanchez-Camacho and Bovolenta, 2008). While Shh signaling at the level of the chiasm seems to be important for the inhibitory axon guidance of ipsilateral axons (Sanchez-Camacho and Bovolenta, 2008; Fabre et al., 2010), the activation of Boc in the retina influences the specification of ipsilateral RGCs. In the absence of Boc, ipsilateral RGCs do not differentiate properly and consequently there is a timing-dependent reduction in the number of RGCs positive for Zic2 and a concomitant increase in the number of RGCs expressing Isl2 in the ventrotemporal retina (Sanchez-Arrones et al., 2013). It is possible that Boc, similar to its homolog CDO, acts as a sink for Shh proteins (Cardozo et al., 2014), protecting mouse ipsilateral projecting RGCs from Shh activity, ensuring they adopt the correct identity.

Zic2 and EphB1 expression are both altered in mice carrying mutations in *teneurin2* (*ten2*; Young et al., 2013). Teneurins (Ten-m/Odz) are a family of type II transmembrane proteins that mediate adhesion by homophilic recognition (Tucker and Chiquet-Ehrismann, 2006; Tucker et al., 2011). Another member of the Ten-m family, Ten-m3, has been proposed to regulate the generation of binocular visual circuits in mice (Leamey et al., 2007; Dharmaratne et al., 2012). Mice that lack Ten-m3 show abnormalities in mapping of ipsilateral projections that lead to defects in binocular map alignment and deficits when performing visually mediated behavioral tasks. More recently, a role for Ten-m3 in RGC specification and lamination has been reported (Antinucci et al., 2013). Whether this role of Ten-M3 in retinal connectivity underlies the visual defects observed in the mutant mice remains to be established.

Nogo, a potent neurite outgrowth inhibitor expressed by midline radial glia may also help guide axons into the ipsilateral pathway. Blocking Nogo receptor (NogoR) function in a semi-intact preparation of the developing visual system results in a reduction in the size of the ipsilateral projection. No effect was observed on the contralateral projection after Nogo downregulation *in vivo*, but *in vitro*, outgrowth of both presumptive ipsilateral and contralateral axons was inhibited (Wang et al., 2008). NogoR is expressed in both ipsilateral and contralateral RGCs, and because it is difficult to distinguish between axons that had navigated through the chiasm before versus during the treatment period, it is possible that an impact on the contralateral projection was just not detected. Another possibility is that Nogo receptors are downregulated on the commissural, but not ipsilateral, growth cones when they approach the midline. Further studies will be required to clarify the role of Nogo signaling in optic chiasm development.

### Factors that Impact RGC Trajectories Through Patterning of the Retina and Ventral Diencephalon

In addition to Zic2 and Isl2, mutations in a number of other patterning genes have been reported to impact growth and navigation of RGC axons at the optic chiasm. These RGC axon pathfinding defects are generally related to changes in the specification and patterning of the retina and ventral diencephalon rather than a direct effect on axon growth or guidance. This is the case for zebrafish *belladona/lhx2* mutants. In these mutants, RGCs axons fail to cross the midline due to defects in the organization of the midline glia, with the expression of *Sema3d* being absent from the diencephalon. In addition, expression of *Slit2* is expanded across the midline (Seth et al., 2006).

Other factors such as *foxg1* and *foxd1* play a dual role in patterning both the retina and ventral diencephalon. *Foxg1* and *foxd1* are expressed in nasal and temporal retina, respectively, as well as in complementary patterns in the ventral diencephalon. In mice lacking *foxg1*, ipsilateral determinants such as Zic2 and EphB1 are expressed ectopically in nasal retina. Patterning of the ventral diencephalon is also abnormal and results in the loss of growth-promoting activity from chiasm cells *in vitro*. Due to a combination of these retinal and diencephalic changes, the proportion of RGC axon projecting ipsilaterally is increased substantially in *foxg1* mutants (Pratt et al., 2004; Tian et al., 2008). In mice lacking *foxd1*, RGC axons make a range of pathfinding defects at the optic chiasm, including increased ipsilateral projections, stalling at the midline, and impaired growth into the optic tracts. Surprisingly, given the larger ipsilateral projection, expression of ipsilateral determinants such as *Zic2* and *ephb1* is decreased in the ventrotemporal retina of *foxd1* mutants. The altered routing of RGC axons at the chiasm therefore results predominately from changes in guidance cue expression in the ventral diencephalon (Herrera et al., 2004). More recently, microRNAs have been demonstrated as important for both eye development and patterning of the ventral diencephalon. Dicer is required for production of mature microRNAs. In conditional mutants lacking Dicer from the retina and ventral diencephalon, the eye and optic chiasm develop abnormally. Conditional dicer mutants have microphthalmia due to changes in progenitor cell proliferation, increased apoptosis, and defective ciliary body development (Pinter and Hindges, 2010; Davis et al., 2011). Although patterning of the retina and specification of ipsilaterally versus contralaterally projecting RGCs appears to occur normally in Dicer mutants, RGC axons make pathfinding errors at the midline, including aberrant ipsilateral projections, dispersed growth, and extension into the contralateral optic nerve (Pinter and Hindges, 2010). The downstream microRNA targets important for chiasm development have not yet been identified but may include many of the guidance cues and receptors outlined above.

## Axon Navigation Through the Optic Tracts and Target Recognition

Once RGC axons exit the chiasm, they extend in the optic tracts dorsocaudally over the surface of the diencephalon toward their major targets—the LGN and the SC in mammals and the tectum in lower vertebrates. Although in all species RGC axons are positioned adjacent to the telencephalon as they extend through the optic tracts, they never normally invade this tissue. Similar to its function at the chiasm, Slit-Robo signaling plays an important barrier function that helps define the precise path followed by the RGC axons as they extend through the optic tracts. *Slits* are expressed in the diencephalon and telencephalon bordering the optic tracts (Ringstedt et al., 2000; Hutson and Chien, 2002; Thompson et al., 2006a). In mouse or zebrafish mutants lacking Slit-Robo signaling, many axons stray away from their normal pathway, extending into the telencephalon, other aberrant brain regions, such as the pineal, and across the dorsal midline (Fricke et al., 2001; Thompson et al., 2006a; Plachez et al., 2008). β1-integrin/N-cadherin, known regulators of Slit-Robo signaling (Rhee et al., 2002; Stevens and Jacobs, 2002), are also important for restricting RGC axons to the optic tracts (Stone and Sakaguchi, 1996). However, whether Slits-Robos/β1-integrin/N-cadherin function together or independently in terms of RGC guidance in the optic tracts is not known currently. Other inhibitory factors that help restrict the RGC axons to the optic tracts have also been identified, including Shh (Gordon et al., 2010), CSPGs (Ichijo and Kawabata, 2001; Walz et al., 2002), and Tenascin-R (Becker et al., 2003). Blocking the expression or function of any of these molecules *in vivo* results in RGC axons straying away from the optic tracts. Sfrp-1 also has been implicated in directing growth within the optic tracts. Sfrp-1 is expressed in the region of the developing optic tract and can act directly on RGC axons, in a cAMP and G-protein dependent manner, to both promote and inhibit axon outgrowth. Overexpression of Sfrp-1 in *Xenopus* results in severe optic tract pathfinding errors, including axons extending away from the tract, and aberrant changes in the direction of growth (Rodriguez et al., 2005).

FGF signaling also plays an important role in guiding RGC axons toward and into their targets. FGF-2 can act directly on RGC axons to promote axon outgrowth (McFarlane et al., 1995). *Xenopus* RGCs expressing dominant-negative FGFRs grow slower than normal and bypass rather than enter the tectum (McFarlane et al., 1996). FGF signaling also acts indirectly to control optic tract development through regulating the expression of other guidance signals. In *Xenopus*, *slit1* and *sema3a* are expressed in the mid-diencephalon, in the region where RGC makes a caudal turn toward the tectum (Campbell et al., 2001; Atkinson-Leadbeater et al., 2010). Inhibiting FGFR function in the optic tract neuroepithelium induces a rapid downregulation of *slit1* and *sema3a* expression levels, and failure of RGC axons to extend beyond the mid-diencephalon. Knockdown of both *slit1* and *sema3a*, but not either alone, induces similar RGC axon guidance defects to loss of FGF-signaling in the neuroepithelium (Atkinson-Leadbeater et al., 2010). Target recognition may also be dependent on sphingosine 1-phosphate (S1P) signaling in RGC growth cones (Strochlic et al., 2008). S1P is expressed around the developing *Xenopus* optic tract and can act directly on RGC growth cones to induce repulsive responses. Blocking S1P synthesis in the region of the developing optic tract results in many axons bypassing the tectum.

HSPGs are key modulators of Slit-, FGF-, and S1P-signaling and play important roles in optic tract development. Addition of exogenous HSs, heparitinase treatment, or inhibiting sulfation of endogenous HSs induces defects in RGC axon extension and tectal bypass defects (Walz et al., 1997; Irie et al., 2002). HSPGS have also been implicated in controlling the sorting of RGC axons prior to entering their targets. Dorso-ventral segregation and age-related ordering of RGC axons in the optic tract of zebrafish and mammals has been reported (Scholes, 1979; Chan and Guillery, 1994; Colello and Guillery, 1998; Lee et al., 2004). In zebrafish *dak/ext2* and *box/extl3* compound mutants with disrupted HS biosynthesis, dorsal RGC axons fail to sort normally (Lee et al., 2004). Segregation of dorsal and ventral axons occurs independently of Slit-Robo signaling (Lee et al., 2004) but requires HSPG-dependent axon degeneration to eliminate missorted axons (Poulain and Chien, 2013). Whether FGF- or S1P-signaling is important for axon sorting has not been established.

Although in mouse and zebrafish NRP1 localizes to RGC axons from early developmental stages, playing a role in accurate guidance at the chiasm (Erskine et al., 2011; Dell et al., 2013), *Xenopus* RGC growth cones only express *Nrp1* and gain responsiveness to Sema3A at later stages, corresponding to when they reach the optic tracts (Campbell et al., 2001). The mechanisms underlying this call autonomous change in *Nrp1* expression in *Xenopus* RGCs are beginning to be understood and involve inhibition of CoREST, a repressor of *Nrp1* transcription, by the microRNA miR124 (Baudet et al., 2012). In turn, sema3A can induce local translation in growth cones of the homophilic cell adhesion molecule NF-protocadherin and its cofactor TAF1, important for guidance at the mid-diencephalon caudal turn point (Leung et al., 2013). Thus, *Xenopus* RGC growth cones display highly dynamic responses to guidance signals important for growth along the optic tract, modulated by intrinsic changes in intracellular cAMP levels (Shewan et al., 2002), microRNA-controlled timing of receptor expression (Baudet et al., 2012), and guidance cue-induced local protein translation (Leung et al., 2013).

Some of the molecular mechanisms controlling the axonal targeting to non-image forming nuclei in mammals are now starting to be described. For instance, the T-box transcription factor Tbr2 is essential for the development of several RGC types that participate in the intrinsically photosensitive circuit that mediate the pupillary light reflex (Sweeney et al., 2014). The adhesion molecule Cadherin-6 and Reelin signaling are also important for the targeting of RGC projections into other non-image forming nuclei (Osterhout et al., 2011; Su et al., 2011).

## Refinement at Visual Targets

Upon arrival to the SC, which occurs at perinatal stages in rodents, the primary growth cones of RGC axons that enter the target extend posteriorly overshooting the location of their future terminal zone (Nakamura and O’Leary, 1989; Simon and O’Leary, 1992). Interstitial branches then start forming along the length of the axon shaft. Once formed, the branches preferentially extend along the lateromedial axis toward their future terminal zone (Nakamura and O’Leary, 1989; Simon and O’Leary, 1992; Hindges et al., 2002; McLaughlin et al., 2003a). In a second phase, segments of RGC axons that are distal to the appropriate terminal zone are eliminated. Concomitantly, complex arborizations grow at the correct terminal zone. Therefore, although topographic maps and eye-specific layers at the visual targets fully emerge before visual experience begins (Chapman and Stone, 1996; Chapman et al., 1996a; Issa et al., 1999; White et al., 2001), the maturation of topographic and eye-specific maps takes place through a gradual process during the first two postnatal weeks in mouse.

### Molecular Mechanisms Underlying Map Topography and Axonal Arborizations

**Figure 5.**
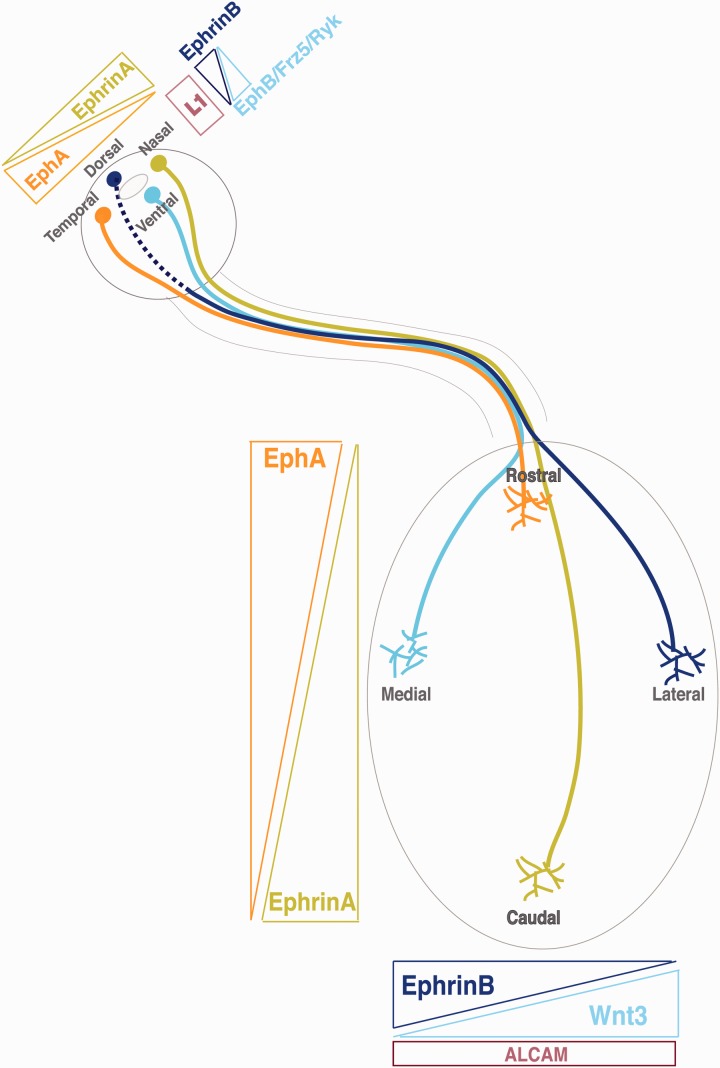
Guidance molecules involved in topographic mapping at the superior colliculus of mice. The retinocollicular connection is highly organized, such that axons from neighboring neurons in the retina terminate in neighboring positions in the superior colliculus. Axons originating in the nasal retina terminate in the caudal SC, while temporal axons go to the rostral SC. In addition, axons originating in the dorsal retina map to the lateral SC and ventral axons terminate in the medial SC. Retinocollicular mapping is mediated by reciprocal molecular gradients in the retina and SC. EphA receptors are expressed by RGCs in an increasing nasal-temporal gradient. Neurons located more temporally express progressively more receptors. Ephrin-A ligands are expressed in the SC in an increasing rostro–caudal gradient. Nasal RGC axons project further into the SC because they express fewer EphA receptors and are subsequently less sensitive to the repulsive ephrin-A ligands. Conversely, the axons of temporal RGCs invade only a short distance into the SC because of their high sensitivity to ephrin-A ligands. In the dorsoventral axis, a gradient of EphB receptors exists in the retina with highest expression ventrally, while a gradient of ephrinB, highest medially, is expressed in the colliculus. In this case, however, EphB/ephrinB signaling seems to mediate attraction. Counterbalancing ephrinB1-EphB activity, a gradient of Wnt3 is highly expressed in the lateral SC and repels ventral axons expressing Ryk. Frizzled, also expressed by ventral axons, mediates attraction to medial SC cells expressing low levels of Wnt3. All RGC axons express the cell adhesion molecule L1-NCAM which, in combination with EphB/ephrinB signaling, seems to play a role in lateromedial mapping by interacting with ALCAM.

Evidence from independent studies has conclusively demonstrated that the major players controlling the formation of retinotopic maps in the main visual targets are the Ephs and ephrins (Figure 5). EphrinAs and ephrinBs are expressed in both the retina and the visual targets. RGCs express EphA receptors in a high-temporal to low-nasal gradient, whereas ephrinA ligands, which are attached to the cell membrane via a glycosylphosphatidylinositol anchor, are highly expressed in the caudal SC and low in rostral collicular areas. Axons that express high levels of EphAs are unable to terminate in the posterior SC because of the repelling ephrins forcing them to terminate in the anterior SC (Frisen et al., 1998; Feldheim et al., 2000; Carreres et al., 2011). Ectopic expression of the receptor EphA3 in the *Isl2* locus demonstrated that rostrocaudal topographic mapping is generated by relative rather than absolute levels of EphA receptors in RGCs (Brown et al., 2000).

The repulsive activity of collicular ephrinA5 to temporal axons seems to be potentiated by the activity of the homeobox transcription factor Engrailed (Brunet et al., 2005). Engrailed is expressed in the SC (Wizenmann et al., 2009) and stimulates the adenosine A1 receptor (A1R) present at higher concentrations in temporal rather than nasal growth cones. A1R is coupled to G proteins and its stimulation by Engrailed inhibits the activity of Adenylate Cyclase 1 (AC1; Stettler et al., 2012), which is required for the emergence of focused terminal zones and elimination of inaccurately targeted collaterals at the level of individual axons (Dhande et al., 2012). AC1 activation drops cAMP accumulation and stimulates rapid synthesis and secretion of ATP from the growth cones. In agreement with these observations, *in vitro* assays have also shown that cAMP oscillations modulate rostrocaudal topographic mapping (Nicol et al., 2007).

EphrinAs are also expressed in the retina in a high-nasal low-temporal gradient (Marcus et al., 1996) and conversely EphAs are expressed in the SC in a pattern reversely oriented to the ephrinAs gradient (Rashid et al., 2005). Reverse signaling with retinal ephrinA acting as a receptor and collicular EphA as the ligand may play a role in the establishment of topography. In agreement with this idea, overexpression of ephrinA5 in the central retina leads to misprojection of these neurons to the caudal SC (Carreres et al., 2011). Conversely, topographic mapping is disrupted in mutant mice null for EphA7, which is expressed in the SC but not in the retinal tissue (Rashid et al., 2005), and an increase in the levels of EphAs in the SC provokes a misprojection of nasal axons to more posterior positions (Yoo et al., 2011). EphrinAs do not posses an intracellular domain, but they are able to trigger intracellular signaling through association with other transmembrane proteins. For instance, the neurotrophin BDNF promotes interstitial branching *in vitro* through a mechanism dependent on TrkB (Marler et al., 2008). EphrinAs on RGC axons also interact with another BDNF receptor, p75 (Lim et al., 2008). The precise function of p75 is unclear, but it is likely that its activation does not reduce branching per se but rather inhibits BDNF/TrkB signaling. Additionally, BDNF controls axon branching of RGCs via the microRNA miRNA-132, which downregulates p250GAP, a GTPase-activating protein that suppresses Rac function. Therefore, BDNF signaling may well control both global branching competence and local branch formation using miRNA-132-dependent and -independent pathways respectively (Marler et al., 2013).

It has also been proposed that retinal ephrinA desensitizes the EphA receptors through cis interactions in the same cells, creating a decreasing temporal-to-nasal gradient of *sensitive* EphA receptors (Hornberger et al., 1999; Carvalho et al., 2006). This cis interaction could create a gradient for the two EphA receptors—EphA3 in the chick and EphA4 in the mouse—that instead of being expressed in a gradient are homogeneously detected in the RGC layer (Feldheim et al., 1998; Brown et al., 2000; Reber et al., 2004). These two mechanisms proposed for the function of retinal ephrinAs—reverse signaling and desensitization of EphA receptors—may not be mutually exclusive and could be acting in parallel during the establishment of topographic maps.

EphBs/ephrinBs are implicated in setting up mapping along the lateromedial collicular axis (Hindges et al., 2002; Mann et al., 2002; Thakar et al., 2011; Figure 5). EphB1/B2/B3 receptors are all expressed in an increasing dorsal-to-ventral pattern in the retina, while ephrinB1/B2 are expressed in an increasing lateromedial gradient in the SC. The individual role of each EphB receptor in the retina is similar, and it has been shown that the overall level of EphBs is more important than EphBs identity in the establishment of lateromedial mapping (McLaughlin et al., 2014). Because ventral EphBs-expressing RGCs project to the medial SC that expresses ephrinBs, it has been hypothesized that ephrinBs attract RGC axon interstitial branches (Hindges et al., 2002). An attractive response mediated by EphB/ephrin binding has, however, never been clearly demonstrated. At the same time as mediating attraction, EphB/ephrinBs may act bifunctionally as branch repellents when expressed at high levels (McLaughlin et al., 2003b). Similar to ephrinAs, ephrinBs are expressed in the retina and ephrinB/EphB, reverse signaling also contributes to lateromedial mapping at the SC (Hindges et al., 2002; Thakar et al., 2011).

Other factors, acting either to modulate ephrinB signaling or in parallel pathways also are important for lateromedial mapping. The interaction of the cell adhesion molecule L1 with the cytoskeletal adaptor ankyrin interferes with Eph signaling during lateromedial mapping. Mice carrying mutations in the L1 ankyrin-binding motif display abnormal lateromedial mapping of RGC axons, resembling the phenotype of EphB mouse mutants. In fact, EphB regulates L1 phosphorylation during lateromedial retinocollicular map formation (Dai et al., 2012). The activated leukocyte cell adhesion molecule (ALCAM) is expressed in the SC during RGC axon targeting, and *in vivo* experiments suggest a model in which ALCAM in the SC interacts with L1 on RGC axons to promote medial extension of RGC axon branches (Buhusi et al., 2009).

Wnt3 is highly expressed in medial areas of the SC, similar to the expression pattern of ephrinB1. The Wnt receptor Ryk, which is highly expressed in the ventral mouse retina, induces repulsion of ventral axons to Wnt3. In addition, Wnt3 attracts dorsal RGC axons at very high concentrations. Wnt3-induced stimulation seems to be mediated by the Frizzled-5 receptor, expressed in the RGC layer in a homogeneous manner (Schmitt et al., 2006). The current model proposes that repulsive Wnt-Ryk signaling competes with the attractive Wnt-Frizzled interaction to generate the response to graded Wnt3 expression.

The graded expression of EphA/Bs and ephrinA/Bs along the temporal—nasal and dorsal—ventral retinal axis required to define specific projection patterns and arborization at the targets result from the action of factors that define retinal polarity at early steps of retinal development. As previously mentioned, the transcription factors Foxg1 and Foxd1 specify nasal and temporal retinal identity, respectively. In addition to being essential for the proper expression of Zic2 and EphB1, Foxg1 and Foxd1 are required for the later gradual expression of retinal EphA/ephrinAs (Herrera et al., 2004; Pratt et al., 2004; Tian et al., 2008; Carreres et al., 2011). Bone morphogenetic proteins (BMPs), in particular BMP4, define retinal polarity by controlling the expression of the T-Box transcription factors Tbx5 and Tbx2 (Chapman et al., 1996b; Sowden et al., 2001; Behesti et al., 2006), and Vax2, that are important for specifying dorsal and ventral retinal identity, respectively (Koshiba-Takeuchi et al., 2000; Barbieri et al., 2002; Mui et al., 2002; Gross and Dowling, 2005). Loss-of-function studies in mice demonstrate that Vax2 is required for the expression of EphA5, EphBs, and ephrinBs along the dorsoventral axis of the retina, resulting in retinotectal axons misrouted along the lateromedial axis of the SC (Barbieri et al., 2002; Mui et al., 2002). Misexpression of Tbx5 induces the expression of ephrinB1/B2 in the ventral half of the retina, leading to misprojection of RGC axons in the targets (Koshiba-Takeuchi et al., 2000).

### Mechanisms Underlying Axonal Refinement

Upon the formation of a rough map controlled by the molecular mechanisms described above, the system undergoes a more local refinement process modulated by what is known as spontaneous retinal waves. Around birth, spontaneous electric waves are initiated by a class of cholinergic interneurons in the retina: the starburst amacrine cells. These cells spontaneously depolarize approximately every 15 s causing a large Ca^2+^ influx that leads to the activation of neighboring RGCs (Feller et al., 1996; Zheng et al., 2004). A wave is initiated when a certain number of amacrine cells become active in a particular area of the retina. As the mouse visual system matures, from P10 to P14, retinal waves change to being mediated by ionotropic glutamate receptors (Wong et al., 1993; Bansal et al., 2000; Demas et al., 2003; Syed et al., 2004). This switch from acetylcholine to glutamate transmission is a gradual process and depends on the maturation of bipolar cells, the main glutamate source in the retina (Miller et al., 1999).

In mice, the onset of cholinergic retinal waves during the first week after birth coincides with axonal remodeling during map formation. This, together with the initial observation that pharmacological and genetic blockade of retinal electric activity blurs mapping at the SC (O’Leary et al., 1986; Stellwagen and Shatz, 2002; McLaughlin et al., 2003a; Chandrasekaran et al., 2005; Dhande et al., 2011), led to the idea that EphA/ephrinA signaling could be influenced by retinal spontaneous activity (Nicol et al., 2007). However, recent experiments using transgenic mice with altered patterns of retinal activity showed a nearly normal rostrocaudal topography (Xu et al., 2011), indicating that large-scale topography is independent of patterned spontaneous retinal activity. This finding has been confirmed molecularly by the demonstration that EphA/ephrinA repulsive signaling acts even in the absence of retinal activity (Benjumeda et al., 2013).

Spontaneous activity is however essential for fine-scale axonal remodeling including eye-specific refinement (Shatz and Sretavan, 1986; Penn et al., 1998; Cook et al., 1999; Rossi et al., 2001; Stellwagen and Shatz, 2002; Xu et al., 2011). RGC arborizations from the two eyes initially overlap and subsequently segregate into nonoverlapping eye-specific territories (Shatz and Sretavan, 1986). Retinal waves preferentially originate in the binocular region (the ventrotemporal retina in the mouse) and propagate, following a biased direction, toward the dorsal-nasal retina (Ackman et al., 2012). Ipsilateral axons seem to be more susceptible to the effects of the binocular competitive process than contralateral axons. Ventrotemporal RGC axons lacking EphB1 project aberrantly to the opposite side, but segregate from the normal contralaterally projecting axons in a manner that depends on activity (Rebsam et al., 2009). This segregation of ipsilateral axons on the contralateral side may reflect the differential expression of particular proteins such as the serotonin transporter (Sert) in ipsilateral but not in contralateral RGCs (Garcia-Frigola and Herrera, 2010). The influence of spontaneous activity on serotonin signaling during eye-specific segregation is not well understood. It has been proposed that the serotonin receptor 5HT_1B_, expressed in all retinal axon terminals (Upton et al., 1999; Salichon et al., 2001) and coupled negatively to AC1 via G-proteins, is activated by serotonin to inhibit cAMP production (Bouhelal et al., 1988) and calcium entry in contralateral axon terminals. However, ipsilateral RGC axons that express Sert would internalize extracellular serotonin to relieve 5HT_1B_-mediated inhibition, producing cAMP and allowing Ca^2+^ influx. This would promote the retraction of ipsilateral termini allowing eye-specific refinement/segregation.

It has been assumed for years that, after occupancy of their respective territories at the visual targets, the maintenance and stabilization of ipsi- and contralateral terminals at the targets are mediated by glutamatergic transmission (Penn et al., 1998; Stellwagen and Shatz, 2002; Demas et al., 2003). Surprisingly, following conditional removal of the vesicular glutamate transporter 2 (vGlut2) in ipsilateral RGCs, ipsilateral terminals are not prevented from targeting into the appropriated region of the LGN, although there is an abnormal persistence of competing contralateral eye axons in the ipsilateral eye territory. This suggests that, although vGlut2-dependent transmission is important for some aspects of eye-specific refinement, it is not essential for the maintenance and consolidation of axonal territories at the targets (Koch and Ullian, 2010).

In summary, the initial rough connectivity that links RGC axons to their target tissues, delineating central anatomical features of visual circuitry such as retinotopy and eye-specific sorting and regionalization, rely on the action of an intricate network of molecular interactions that coordinate axonal behavior. Retinal spontaneous activity occurring before eye opening make these rough topographic maps more precise and ready to be fully functional at the time of eye opening. It is truly remarkable that, as has been demonstrated beautifully (Maiorano and Hindges, 2013), this sequence of molecular and activity-dependent events confers the developing visual system an extremely plastic capacity to maximize the visual field and its coverage in the brain in response to perturbations such as degeneration.

## Visual Mapping Defects Related to RGC Misrouting at the Optic Chiasm: Albinism and Achiasmia

**Figure 6.**
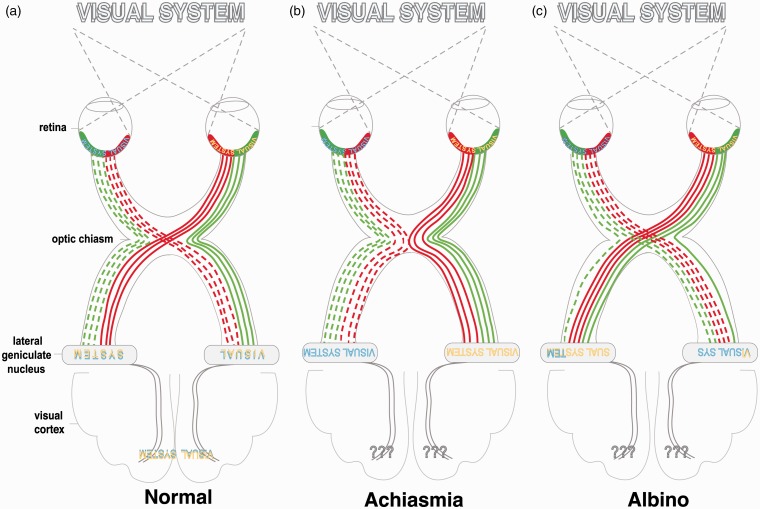
Thalamic defects caused by the misrouting of retinal fibers at the optic chiasm. (a) In primates, nasal RGC axons (red) cross the midline at the optic chiasm while temporal RGC axons (green) extend ipsilaterally, projecting toward the lateral geniculate nucleus and then the visual cortex, maintaining the strict retinotopic segregation of the visual field. (b) In achiasmatic individuals, nasal RGC axons fail to decussate adequately at the chiasm, instead projecting ipsilaterally toward the lateral geniculate nuclei together with temporal RGC axons. (c) In albinism, ipsilaterally destined temporal RGC axons erroneously decussate and project contralaterally at the optic chiasm. In both of these conditions, the corresponding visual field is a complete (achiasmatic) or partial (albinism) mirror inversion of the representation in each hemisphere, creating an incongruent representation that results in a lack of binocular vision and nystagmus. The abnormal visual input perceived in achiasmia or albinism does not induce topographic reorganizations of the thalamocortical projections. Reorganization of local intracortical architecture in the cortex rather underlies the ability to cope with abnormal visual inputs derived from axonal misrouting at the chiasm. N = nasal retina; T = temporal retina.

Albino and achiasmatic conditions are two examples of congenic anomalies with alterations in the number of ipsilateral and contralateral RGC axons that have been analyzed in humans. In albinism, a significant number of temporal retinal fibers erroneously decussate and project contralaterally. Conversely, in achiasmia, nasal retinal fibers fail to decussate, projecting instead ipsilaterally toward the LGN along with temporal retinal fibers. In both cases, the corresponding visual field is completely (achiasmatic) or partially (albinism) reversed in the laminae of the thalamus (Apkarian et al., 1994, 1995; Victor et al., 2000; Prakash et al., 2010). These axonal disarrangements pose a substantial challenge to the organization of visual fields maps at the visual targets and generate potential sensory conflicts (Figure 6). Functionally, an aberrant ratio of ipsilateral/contralateral projections results in lack of stereopsis and nystagmus in the horizontal plane or seesaw nystagmus in the vertical plane (Williams et al., 1994; Dell’Osso et al., 1998; Apkarian and Bour, 2001; Huang et al., 2006; von dem Hagen et al., 2008; Hoffmann et al., 2012).

At the molecular level, albinism is caused by alterations in the expression levels of the *tyrosinase* gene or other components of the melanin biosynthetic pathway (Martinez-Garcia and Montoliu, 2013; Kamaraj and Purohit, 2014). Some studies have associated the lack of the melanin precursor DOPA with alterations in the cell cycle of retinal cells (Ilia and Jeffery, 1999; Kralj-Hans et al., 2006). In fact, spatiotemporal aspects of neurogenesis seems to be altered in albino mice (Rachel et al., 2002; Bhansali et al., 2014), and it has been proposed that such alterations could influence the specification of RGCs. In agreement with this, albino mice show a decrease in number of Zic2-/EphB1-positive cells (Herrera et al., 2003; Rebsam et al., 2012) that is compensated by an increase in the number of Isl2 cells in the VT retina during development (Bhansali et al., 2014). However, further experiments are needed to fully understand the mechanisms relating the melanin pathway with retinal axon misrouting at the midline. The molecular alterations underlying achiasmia are not known, but it is possible that mutations in genes such as Lhx2, Fgf8, or Shh, important for establishing the patterning of the optic chiasm region, are the origin of this condition. In fact, *belladona* zebrafish mutants (Lhx2 mutants) exhibit a phenotype similar to achiasmatic individuals with misrouting of retinal axons to the ipsilateral side of the brain accompanied by congenital nystagmus (Huang et al., 2006).

At the LGN, misrouted axons from the VT retina of albino mice project within the contralateral LGN forming a patch separated from the contralateral termination area (Rebsam et al., 2012). This result confirms the result from EphB1 mutants, demonstrating that originally ipsilateral axons segregate from the normal contralateral RGCs even when projecting to the same side, suggesting specific refinement mechanisms for ipsi- and contralateral axons. In a canine model of achiasmia, the abnormal ipsilaterally directed nasal fibers innervate the LGN as if they had successfully crossed the midline, terminating in the appropriate layer of the nucleus. As a consequence, the LGN contains noncongruent, mirror-image maps of visual space in adjacent layers also leading to nystagmus (Williams et al., 1994).

The abnormal visual input perceived by achiasmatic or albino individuals does not induce a topographic reorganization in the thalamocortical projection or the occipital callosal connections. Instead, albino and achiasmatic individuals present atypical organization of the visual cortex consisting of overlapping visual hemifield maps with bilateral population receptive fields (Hoffmann et al., 2012; Kaule et al., 2014). Thus, reorganization of local intracortical architecture in the visual cortex seems to underlie the ability to cope with abnormal visual inputs resulting from axonal defects in the retinogeniculate pathway (Figure 6).

## Conclusion

It is clear from the studies discussed in this review that the wiring together of the developing visual system involves a complex interplay of genetic, molecular and activity based mechanisms. Visual function is critically dependent on the correct specification and generation of RGCs, and appropriate guidance of their axons to visual target regions in the brain. Guidance of RGC axons is not a simple process but requires integrated interactions between multiple coexpressed signals and modulatory factors, as well as regulation of intrinsic changes in growth cone responses and guidance cue expression. Once axons reach their targets, their task is far from complete, and molecular mechanisms act in concert with spontaneous activity to induce rearrangement and refinement of axon termini. Disruption of any of these processes can result in severe visual impairment. Although our understanding of the mechanisms controlling visual system wiring has increased substantially over the past 10 to 20 years, much still remains to be established and will form a significant remaining challenge in the years to come.
